# Supplementary material for: Silane Modification of Mesoporous Materials for the Optimization of Antiviral Drug Adsorption and Release Capabilities in Vaginal Media
Source: Pharmaceutics. 2021 Sep 7;13(9):1416. doi: 10.3390/pharmaceutics13091416 (PMC8468001; doi:10.3390/pharmaceutics13091416)
Supplement: Supplementary file 1 [file pharmaceutics-13-01416-s001.zip › pharmaceutics-1339862-supplementary.pdf]

pharmaceutics

MDPI

Supplementary Material: Silane Modification of Mesoporous Materials for the Optimization of Antiviral Drug Adsorption and Releasing Capabilities in Vaginal Media

Elena Whittle, Araceli Martín-Illana, Raul Cazorla-Luna, Fernando Notario-Perez, María Dolores Veiga-Ochoa, Juan Rubio, Aitana Tamayo

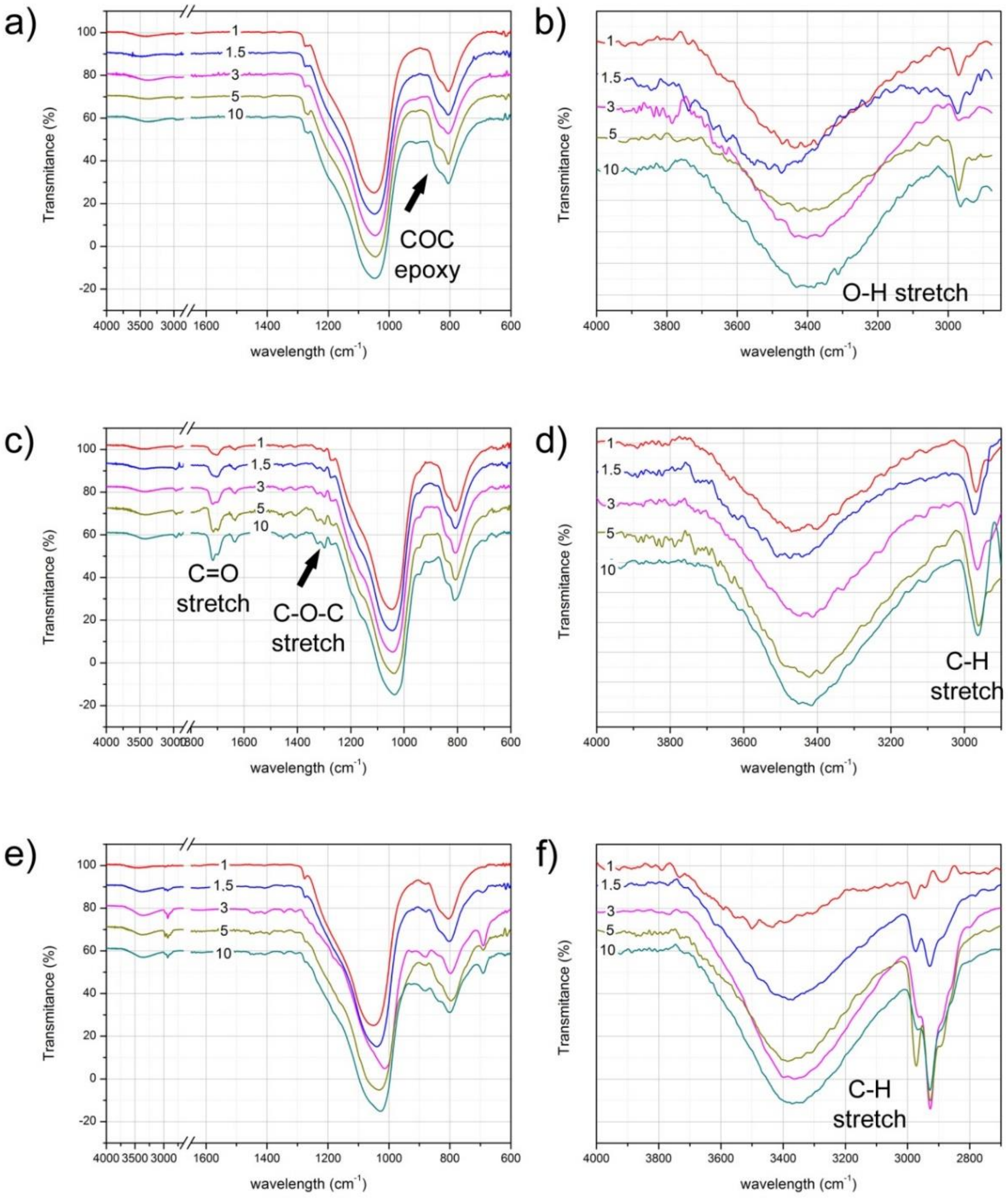

Figure S1. FTIR spectrum of the functionalized mesoporous hybrids **a**) and **b**) Spectra of the materials functionalized with GLYMO (b is a detail of the high-frequency range) **c**) and **d**) Spectra of the materials functionalized with MEMO (d is a detail of the high-frequency range) **e**) and **f**) Spectra of the materials functionalized with MPTMS (f is a detail of the high-frequency range).

Table S1. Table summarizing the fitting constants of the adsorption curves by the application of the Lagergren Model.

		Lagergren model				
		**k_{lag} (min^{-1})**	**C_{∞} (mg)**	**r^2**	**χ^2**	**Prob > F**
Non functionalized		7.245×10^{-2}	11.1	0.880	2.6	7.28×10^{-7}
G	1	1.211×10^{-2}	72.7	0.865	111.6	3.22×10^{-5}
	1.5	6.261×10^{-2}	64.6	0.887	75.1	2.50×10^{-6}
	3	7.910×10^{-3}	85.9	0.953	44.4	5.58×10^{-7}
	5	9.380×10^{-3}	77.6	0.886	104.4	2.30×10^{-5}
	10	1.425×10^{-1}	15.3	0.699	11.0	4.71×10^{-5}
M	1	6.290×10^{-3}	52.4	0.863	44.0	3.28×10^{-5}
	1.5	4.700×10^{-3}	37.4	0.894	16.0	1.61×10^{-5}
	3	3.504×10^{-2}	47.8	0.967	13.5	6.03×10^{-8}
	5	1.301×10^{-1}	32.6	0.462	96.1	4.74×10^{-4}
	10	3.440×10^{-3}	84.6	0.969	23.4	2.46×10^{-7}
S	1	1.135×10^{-2}	37.2	0.672	50.3	2.39×10^{-4}
	1.5	1.796×10^{-1}	34.1	0.676	39.2	1.19×10^{-5}
	3	1.639×10^{-2}	85.9	0.888	130.3	1.08×10^{-5}
	5	1.323×10^{-2}	37.8	0.841	37.1	5.94×10^{-5}
	10	1.323×10^{-2}	53.6	0.841	74.4	5.94×10^{-5}

Table S2. Table summarizing the fitting constants of the adsorption curves by the application of the Pseudo second Model.

		Pseudo-second order model				
		**k_{2s} (min^{-1})**	**C_∞ (mg)**	**r^2**	**χ^2**	**Prob > F**
Non functionalized		7.840×10^{-3}	12.0	0.931	1.50	6.40×10^{-8}
G	1	1.425×10^{-1}	15.3	0.699	11.00	4.71×10^{-5}
	1.5	1.700×10^{-4}	82.9	0.924	62.62	3.43×10^{-6}
	3	1.180×10^{-3}	69.9	0.895	70.33	1.94×10^{-6}
	5	1.011×10^{-4}	96.1	0.952	45.68	6.27×10^{-7}
	10	1.200×10^{-4}	89.2	0.938	56.83	2.17×10^{-6}
M	1	1.373×10^{-4}	58.9	0.914	27.54	5.37×10^{-6}
	1.5	1.504×10^{-4}	41.7	0.932	10.29	2.86×10^{-6}
	3	9.591×10^{-4}	51.5	0.963	14.86	8.83×10^{-8}
	5	3.130×10^{-3}	37.0	0.600	71.46	1.57×10^{-4}
	10	3.650×10^{-5}	100.6	0.977	17.38	7.64×10^{-8}
S	1	4.766×10^{-4}	39.5	0.759	36.96	7.47×10^{-5}
	1.5	9.590×10^{-3}	35.1	0.779	26.78	2.69×10^{-6}
	3	2.234×10^{-4}	62.3	0.896	48.48	1.15×10^{-5}
	5	3.160×10^{-4}	44.0	0.896	24.17	1.15×10^{-5}
	10	2.051×10^{-4}	96.4	0.930	81.83	1.76×10^{-6}

Table S3. Table summarizing the fitting constants of the adsorption curves by the application of the Pseudo second Model.

Elovich model						
		**a**	**b**	**r^2**	**χ^2**	**Prob > F**
Non functionalized		4.54	0.59	0.957	0.93	7.64×10^{-9}
G	1	7.61	0.09	0.937	52.00	1.66×10^{-6}
	1.5	33.08	0.11	0.835	110.32	1.12×10^{-5}
	3	6.39	0.08	0.846	145.87	5.71×10^{-5}
	5	6.21	0.08	0.943	51.98	1.53×10^{-6}
	10	14.71	0.45	0.963	1.34	1.21×10^{-8}
M	1	4.23	0.14	0.895	33.77	1.19×10^{-5}
	1.5	2.78	0.21	0.876	18.78	2.96×10^{-5}
	3	9.77	0.13	0.904	39.08	3.98×10^{-6}
	5	23.80	0.20	0.859	25.19	2.85×10^{-6}
	10	4.00	0.10	0.790	160.26	4.15×10^{-4}
S	1	19.23	0.25	0.847	23.51	1.32×10^{-5}
	1.5	337.12	0.29	0.795	24.79	1.99×10^{-6}
	3	24.16	0.09	0.827	200.99	5.72×10^{-5}
	5	8.42	0.21	0.801	46.36	1.38×10^{-4}
	10	11.92	0.15	0.801	92.98	1.38×10^{-4}

Table S4. Table summarizing the kinetical data obtained through the application of the first order model.

First order model						
		**C (mg)**	**k_{1st} (min^{-1})**	**r^2**	**χ^2**	**Prob > F**
Non functionalized		0.98	5.201×10^{-02}	0.990	1.40×10^{-3}	5.61×10^{-8}
G	1	1.78	9.264×10^{-2}	0.981	8.82×10^{-3}	1.87×10^{-7}
	1.5	0.15	5.268×10^{-2}	0.963	1.13×10^{-4}	2.16×10^{-6}
	3	0.63	7.258×10^{-2}	0.976	1.38×10^{-3}	4.86×10^{-7}
	5	2.57	8.145×10^{-2}	0.975	2.36×10^{-2}	4.59×10^{-7}
	10	0.15	5.341×10^{-2}	0.982	5.81×10^{-5}	2.95×10^{-7}
M	1	0.31	8.069×10^{-2}	0.987	1.82×10^{-4}	6.90×10^{-8}
	1.5	0.42	4.724×10^{-2}	0.987	3.23×10^{-4}	1.32×10^{-7}
	3	1.09	8.648×10^{-2}	0.967	5.65×10^{-3}	9.93×10^{-7}
	5	0.45	7.256×10^{-2}	0.975	7.45×10^{-4}	5.65×10^{-7}
	10	0.24	4.328×10^{-2}	0.978	1.73×10^{-4}	7.22×10^{-7}
S	1	0.76	7.824×10^{-2}	0.970	2.51×10^{-3}	8.59×10^{-7}
	1.5	2.15	5.667×10^{-2}	0.960	2.55×10^{-2}	2.67×10^{-6}
	3	0.52	5.152×10^{-2}	0.980	7.96×10^{-4}	4.93×10^{-7}
	5	0.45	5.590×10^{-2}	0.971	8.47×10^{-4}	1.16×10^{-6}
	10	0.90	6.887×10^{-2}	0.966	3.96×10^{-3}	1.43×10^{-6}

Table S5. Table summarizing the kinetical data obtained through the application of the Korsmeyer-Peppas model.

		Korsmeyer – Peppas model					
		**C (mg)**	**K_{KP} (min^{-1})**	**n**	**r^2**	**χ^2**	**Prob > F**
Non functionalized		1.85	9.254×10^{-2}	0.39	0.941	8.57×10^{-3}	6.80×10^{-5}
G	1	3.30	1.649×10^{-1}	0.28	0.921	3.66×10^{-2}	7.014×10^{-5}
	1.5	0.30	9.915×10^{-2}	0.36	0.982	5.50×10^{-5}	2.611×10^{-6}
	3	1.04	1.492×10^{-1}	0.32	0.953	2.72×10^{-3}	2.406×10^{-5}
	5	4.60	1.535×10^{-1}	0.30	0.948	4.98×10^{-2}	2.745×10^{-5}
	10	0.24	1.181×10^{-1}	0.38	0.961	1.29×10^{-4}	2.162×10^{-5}
M	1	0.50	1.670×10^{-1}	0.30	0.925	1.07×10^{-3}	7.31×10^{-5}
	1.5	0.83	8.298×10^{-2}	0.40	0.961	9.80×10^{-4}	2.40×10^{-5}
	3	2.74	1.142×10^{-1}	0.29	0.959	7.05×10^{-3}	1.41×10^{-5}
	5	1.05	1.051×10^{-1}	0.32	0.956	1.32×10^{-3}	2.12×10^{-5}
	10	0.47	7.755×10^{-2}	0.41	0.979	1.63×10^{-4}	5.38×10^{-6}
S	1	1.80	1.126×10^{-1}	0.30	0.958	3.49×10^{-3}	1.63×10^{-5}
	1.5	4.23	1.058×10^{-1}	0.35	0.944	3.57×10^{-2}	4.29×10^{-5}
	3	0.94	9.444×10^{-2}	0.39	0.956	1.77×10^{-3}	3.33×10^{-5}
	5	0.73	1.215×10^{-1}	0.37	0.972	8.18×10^{-4}	8.48×10^{-6}
	10	1.14	1.897×10^{-1}	0.33	0.967	3.78×10^{-3}	9.90×10^{-6}

Table S6. Table summarizing the kinetical data obtained through the application of the Weibull model.

		Weibull model					
		**C (mg)**	**b**	**a**	**r^2**	**χ^2**	**Prob > F**
Non functionalized		1.03	0.83	13.30	0.994	8.85×10^{-4}	2.37×10^{-7}
G	1	1.86	0.72	6.14	0.992	3.57×10^{-3}	2.11×10^{-7}
	1.5	0.19	0.59	8.73	0.997	9.52×10^{-6}	3.27×10^{-8}
	3	0.69	0.67	7.20	0.995	3.12×10^{-4}	1.09×10^{-7}
	5	2.78	0.66	6.30	0.995	4.34×10^{-3}	6.25×10^{-8}
	10	0.17	0.73	10.54	0.995	1.54×10^{-5}	1.08×10^{-7}
M	1	0.32	0.77	7.61	0.995	7.56×10^{-5}	9.84×10^{-8}
	1.5	0.46	0.77	12.71	0.996	9.71×10^{-5}	7.50×10^{-8}
	3	1.21	0.61	5.57	0.998	3.97×10^{-4}	1.07×10^{-8}
	5	0.50	0.66	7.16	0.995	1.46×10^{-4}	8.62×10^{-8}
	10	0.28	0.68	12.19	0.997	2.52×10^{-5}	5.08×10^{-8}
S	1	0.84	0.63	6.24	0.995	3.76×10^{-4}	6.31×10^{-8}
	1.5	2.39	0.68	9.01	0.971	1.83×10^{-2}	8.13×10^{-6}
	3	0.57	0.74	11.41	0.991	3.66×10^{-4}	6.52×10^{-7}
	5	0.53	0.65	8.99	0.995	1.35×10^{-4}	9.47×10^{-8}
	10	1.04	0.61	6.94	0.995	6.26×10^{-4}	1.11×10^{-7}
